# Prevalence and associated factors of self-treatment behaviour among different elder subgroups in rural China: a cross-sectional study

**DOI:** 10.1186/s12939-020-1148-2

**Published:** 2020-03-12

**Authors:** Wanchun Xu, Zhong Li, Zijing Pan, Ruibo He, Liang Zhang

**Affiliations:** School of Medicine and Health Management, Tongji Medical College, Huazhong University of Science and Technology, No 13 Hangkong Road, Qiaokou District, Wuhan, 430030 Hubei China

**Keywords:** Self-treatment, The elderly, Rural China, Family practice, Empty nest

## Abstract

**Background:**

Self-treatment is a common and widespread behaviour, of which the risks are multiplied in old age. However, the determinants of self-treatment among elders in rural China remain unclear. This study aims to explore the prevalence and associated factors of self-treatment among elders in rural China, trying to discover the vulnerable groups as well as the service gaps among the rural elders.

**Methods:**

Based on a multi-stage stratified random sampling method, a cross-sectional household survey was conducted among 30 villages in Sinan County, an impoverished county in western China. Data were collected through a household–individual combined questionnaires. The analysis was restricted to elders who reported illness within the last 2 weeks, and the final sample size was 330 (individuals). Bivariate and multiple logistic regression analysis were performed in the whole sample group and four subgroups to obtain the prevalence ratios regarding the associated factors.

**Results:**

In the present study, 35.2% of the elders with illness within the last 2 weeks reported self-treatment. The variables associated with self-treatment in the whole sample group were health status (OR 6.75, 95%CI 1.93–23.60), recent alcohol consumption (OR 0.42, 95%CI 0.21–0.83) and the utilisation of family practice services (OR 0.59, 95%CI 0.36–0.96); the same predictors were found in the subgroup of elders with chronic diseases. No significant predictors were found in the subgroup of elders without chronic diseases. Empty-nest elders with higher affinity to traditional Chinese medicine (OR 0.39, 95%CI 0.18–0.86) or drinking alcohol recently (OR 0.28, 95%CI 0.09–0.82) were less likely to self-treat, while the non-empty-nest elders who were no less than 75 years old (OR 3.10, 95%CI 1.33, 7.22) or at better health status (OR 9.20, 95%CI 1.73–48.75) were more likely to self-treat.

**Conclusion:**

Self-treatment was prevalent among the elders in rural China. Better health status, no recent alcohol consumption and no utilisation of family practice are associated with self-treatment among rural elders. Older elders in the non-empty nest group were more likely to self-treat, while the empty-nest elders with self-care habits in traditional Chinese medicine were less likely to self-treat. Deeper understanding of the self-treatment behaviour among rural elders may provide insights for identifying the potential service gaps and developing improvement strategies in the health care delivery system for the elderly in China.

## Background

Progressive ageing has raised a great challenge to the healthcare system in many countries. As the WHO advocated, the fragmented healthcare systems need to be transformed to provide integrated healthcare services to respond to the needs of the elderly [1]. Some developed countries, such as the UK, Australia and Canada, have established relatively complete healthcare delivery systems for the elderly [2]. However, the availability of affordable and convenient medical care to elders in low and middle income countries (LMICs) is still far from being satisfactory [3]. Costly medical expense, inconvenience to medical visits, distrust on the healthcare system and other social changes lead to the non-use of health services or self-treatment behaviour of the elderly [4–6]. Due to the increasing drug availability and the permissive regulation of drug retailing, the latter choice is more common in LMICs, especially in underdeveloped rural areas [7].

Self-treatment refers to ‘the scenario where a person uses unprescribed drugs or other approaches to cope with illness conditions’ [8]. Self-medication is the most common form of self-treatment, which has been widely discussed in the fields of health behaviours and drug management [9, 10]. According to the WHO, self-medicine refers to the use of medicine by individuals to treat self-recognised illness or symptoms [11]. The forms of self-medication included over-the-counter (OTC) medicine use, prescription medication purchased using expired and non-refillable prescriptions, leftover drugs from old prescriptions, prescription medications offered by family and friends, and so on [12]. Besides self-medication, there are other common approaches of self-treatment including massage, physiotherapy and so on.

Self-treatment is highly prevalent among the elderly in some countries. A systematic review revealed that the prevalence of self-medication among elders varies from 20 to 60% in most studies [9]. The data of the China National Health Survey (CNHS) in 2013 suggested that the prevalence of self-treatment among the elderly is 44.2%, with a slight difference between urban (43.1%) and rural (45.8%) areas [13]. Although self-treatment can increase the individuals’ availability of medical help and reduce the pressure on the healthcare system, several risks are involved in self-treatment, such as incorrect self-diagnosis, delays in seeking for professional help and adverse drug reactions (ADRs) due to the inappropriate use of medicine [10, 14]. Some previous studies have shown that elders are at higher risk for illness than other age groups; thus, elders are prone to greater consumption of drugs and higher risk of inappropriate drug use [15]. Moreover, inappropriate use of drug would result in more serious ADRs for the elders due to age-related changes in the pharmacokinetics and pharmacodynamics of drugs [16].

An official or agreed definition of ‘self-treatment’ is still lacking worldwide. The present study is based on the data from a household survey conducted in a poor rural county in western China. The definition of self-treatment in the questionnaires referred to the China National Health Survey (CNHS) and other previous studies. In the CNHS, ‘self-treatment’ is defined as ‘taking some drugs and/or other remedies, or having a massage and/or physiotherapy rather than visiting a physician when experiencing symptoms or complaints during the two weeks preceding the survey’ [17]. Based on the definition of CNHS, self-treatment is defined in the questionnaire of the present study as ‘taking drugs and/or other physiotherapy approaches without physicians’ advice to cope with the symptoms or complaints during the two-week period preceding the survey’. According to the review on previous studies, the behaviours considered as self-treatment in the present study includes purchasing OTC medicine with self-diagnosis from drug stores, using leftover drugs or purchasing drugs from expired prescription, taking the prescription drugs given by the friends or relatives, and receiving acupuncture, massaging or other physiotherapy treatments at home or in non-medical institution.

Previous studies have examined the potential determinants of self-medication and self-treatment behaviour among the elderly. Female sex, visits to pharmacists, depression, functional dependency, recent hospitalisation, oral pain and restriction of physical inactivity are positively associated with self-medication, whereas medical appointments, married status, use of health services, satisfaction with living arrangement, living in institutional settings and private health plans are negatively related to the choice of self-medication [9]. Long-term illness is broadly proven to be associated with the higher possibility of self-medication [12] or self-treatment [17]. The decision to self-treat involves an interaction between internal dynamic and external strengthening mechanisms, including individual, household, accessibility and medical insurance system factors [17]. However, the factors examined in studies with Chinese settings mostly included the socio-demographic characteristics and health status of individuals. The roles of the health-care delivery system and the social changes [5] were rarely given attention. This knowledge gap may result in an incomprehensive understanding of the mechanisms underlying the self-treatment behaviour of the elderly in current China, as well as the identification of the vulnerable groups.

According to the literature review, several factors in the setting of rural China may contribute to the better understanding of the knowledge gaps mentioned above. Firstly, the condition of the family practice system may influence the rural elders’ health-seeking behaviour, including their resorting to self-treatment. The family practice contract service has been implemented in China in the new round of the healthcare reform since 2009, aiming to build a closer relationship between the residents and family practice physicians, which would help doctors improve the efficacy and comprehensiveness of primary care services [18]. However, the effect of the family practice on some of the residents’ health-seeking behaviour in China has not been examined yet. Secondly, as for the social changes, China is experiencing rapid ageing and urbanisation, and the number of empty-nest elders who are living away from their children is increasing in rural areas. The impact of family caregivers on the elders’ medical decision-making has been well documented [19–22]. A study in China indicated that empty-nest seniors have a higher non-use rate of healthcare services [23]. The empty-nest effect on the elders’ self-treatment remains unknown. Thirdly, the role of traditional Chinese Medicine (TCM) is worthy of attention. The TCM theory is often used to guide self-care practices in the daily life of Chinese people [24]. However, research published in both English and Chinese presented a shortage of discussion on the self-treatment issue from the perspective of TCM [25].

In this study, we explored the factors associated with self-treatment among the elderly using the data of a household survey conducted in a poor county in rural western China. The variables related to the knowledge gaps mentioned above were considered in the study design, which distinguishes this study from previous ones. Considering that the long-term illness [26] and condition of the caregivers [21] may influence the elders’ health-seeking pattern, we conducted the analysis separately in the whole sample group and four subgroups (i.e. elders with chronic diseases, elders without chronic diseases, empty-nest elders and non-empty-nest elders). From the perspective of universal health coverage, deeper understandings of the self-treatment issue among rural elders are crucial to discovering not only the potential vulnerable groups who may be at higher risk of inequality to health service access [23], but also the service gaps [17] in the present health care system. These findings will be constructive for the improvement of the healthcare delivery system for the elderly.

## Methods

### Study design and population

This study was based on the data from a national survey to assess the residents’ healthcare needs, which is a part of a research on the capacity-building of the primary care system in China. The national household survey mentioned above was carried out with general population in four counties/districts across China, including the two districts in urban area (Futian in eastern China, Xiling in central China) and two counties in rural area (Dangyang in middle China, Sinan in western China). With 489 administrative villages [27] and the population size of 499,398 [28], Sinan county is an impoverished county on the list of national-level poverty-stricken counties recognised by the State Council of China. Eighteen minority ethnic groups are also living in the county of Sinan [29]. The data collected in Sinan were analysed in this article. The household survey in Sinan was conducted in July 2018.

A multi-stage stratified random sample was drawn to conduct one-to-one interviews, and the design effect was set at 2.5 in the sample size calculation. With an allowable error for significance level of 0.05 and the incidence of chronic diseases among the population (21.338%) according to the fifth CNHS [13], the minimum sample size was calculated to be 3584 individuals in 1235 families in 30 villages in each study centre. In the first stage, five towns were randomly selected according to the distance to the county hospital. In the second stage, six villages were randomly selected in each town according to the distance to the township hospitals. In case of refusal or closed household, as well as the deletion of some defective questionnaires, extra families were included in the sample to make sure that at least 42 families were effectively interviewed in each village. All the sampled families were systematically selected in the rosters of residents from the village councils. All the members of the sampled families were investigated. Finally, 3983 individuals in 1355 households in 30 villages were investigated in Sinan. All the investigators were previously trained. In the quality control procedure, the questionnaires were audited by the experienced supervisors at the end of the investigation every day. The defective questionnaires were complemented by phone, and those failed to be complete were dropped. Referring to the previous studies, our analysis was restricted to the elders (≥60 years old, 1303 individuals in total) who reported the presence of illness during the two-week period before they were interviewed (338 individuals in total). Those who did not answer some of the variables in the present study were excluded from the analysis, and the final sample size was 330 individuals.

This study was approved by the Ethics Committee of Tongji Medical College, Huazhong University of Science and Technology (IORG No: IORG0003571).

### Materials and variables

Data were collected through household–individual combined questionnaires, including basic information of families, individual demographic background, health condition, health habits and utilisation of health care. Those reporting any one of the following situations in the 2 weeks leading up to interview were considered as having illness or discomfort in the previous 2 weeks: 1) visiting the medical institution due to the diseases or injuries; 2) taking drugs or other complementary physiotherapy due to the diseases or injuries; 3) absence from work or study or resting in bed for at least 1 day due to the discomfort [13]. Those who had illness or discomfort but had not taken any actions mentioned in the three categories above were not included in the analysis, such as the elders in stable condition of chronic diseases or those with minor discomfort, who did not need any medical help or rest.

To identify individuals’ self-treatment behaviour, those who had reported illnesses or discomfort in the past 2 weeks were asked to answer the following question: ‘How did you treat your illness in the past two weeks?’ The response options were: 1) self-treatment (self-medicine or other approaches); 2) outpatient visit; 3) hospitalisation; 4) no treatment. The first choice was considered as ‘self-treatment’. Those practised self-medicine but got the medicine from the township hospitals or general hospitals were considered as being under treatment with doctors’ instruction, and were excluded from the subjects of self-treatment. All the respondents were informed of the definition and the specific forms of self-treatment in this study mentioned above, which was written in the booklets of guidance for the investigators. Thus, a new dependent variable was created to determine if a person practises self-treatment (self-treatment versus non-self-treatment).

All socio-demographic variables were dichotomised, including (1) gender (male and female), (2) age (60–74 or ≥ 75), (3) in poverty (yes or no), (4) illiteracy (yes or no) and (5) the condition of family caregivers (empty-nest or non-empty-nest). The identification system of the poor families is well-developed and strict in the welfare system in China [30]. The families with annual household income per capita below the standard of the poverty in local areas, including the vulnerable groups (elders, disabled persons and minors) with neither income nor supporters, will be identified as some kind of ‘poor families’ (‘Dibao Hu’, ‘Wubao Hu’ or ‘Pinkun Hu’) by the local government [31]. These officially-recognised poor family were considered as poverty population in the present study. Empty-nest elders referred to those who live alone (empty-nest singles) or with a spouse (empty-nest couples) [23]. The EQ-5D-3 L (three-level EuroQol five-dimensions) instrument was applied to access the respondents’ health status. The time trade-off (TTO) technique was one of the methods for evaluating the tradeoffs between the quality of life and length of life, which can be used to generate the value set of the EQ-5D-3 L instrument [32]. The time trade-off value for each respondent can be calculated to quantify the respondent’s health status, based on the scores of the five dimensions (mobility, self-care, usual activities, pain/discomfort, and anxiety/depression) in the instrument. The calculating model developed by Liu etc. for Chinese people was used in the present study [33]. The TTO values ranged from − 0.149 to 1, and higher values represent better health status. Presence of chronic diseases was also used as a variable to describe the elders’ health condition. Variables of health habits include (1) self-care in TCM (yes or no) and (2) recent alcohol consumption (yes or no). Self-care in TCM includes massaging, emotional therapy, dietary supplement usage, traditional sports therapy and other supplementary approaches to remain healthy and prevent diseases in daily life as the philosophy of TCM suggests [34]. It should be noted that self-care differed from self-treatment in the present study. Self-treatment was applied to cope with a certain disease that was recorded in the interview, while self-care was practised to promote general health [11]. Recent alcohol consumption refers to drinking alcohol at least once a week in the previous 6 months and more. Variables of the healthcare delivery system include (1) distance to the nearest medical institution (< 1, 2–3, > 3 km) and (2) utilisation of family practice services (yes or no). The utilisation of family practice services refers to the fact that the elders who had signed the contracts with the family doctors in the local primary care system have used one or more services in the contracts, including personal health education and instruction, personal medical care, personal health management in TCM, health management for special groups, referral service and so on.

The untreated rate of the people with illness in the previous 2 weeks, as well as the most frequent self-treated illnesses and the disease-specific self-treatment rate, were also calculated in this study.

### Statistical analysis

Pearson’s chi-square test was conducted in the descriptive analysis of the sample characteristics. The prevalence of self-treatment were estimated for each of the classified variables in the whole sample and four subgroups. The proportion and respective confidence intervals of 95% (95%CI) were reported. Statistical analysis was carried out using bivariate regression analysis, which not only assessed the significant difference but also reported the effect size in the form of prevalence ratios (crude odds ratios). Additionally, five multiple logistic regression models that included all variables were generated to estimate the independent effect of each variable on self-treatment, one for the whole sample and four for subgroups. The adjusted odds ratios, 95% confidence interval (95% CI) and *p*-value were reported. Data were analysed using Stata version 14.0. Statistical significance was set at *P* < 0.05.

## Results

### Characteristics of subjects

The characteristics of subjects were presented in Table 1. Approximately 25.9% of the elders reported the presence of illness within the past 2 weeks before the investigation. The prevalence of self-reported chronic diseases among the elders reporting illness or discomfort in the previous 2 weeks was 74.2%. Nearly half of the subjects (46.4%) were empty-nest elders. Elders with chronic diseases were more likely to have the habits of self-care in TCM in daily life than the elders without chronic diseases (68.2% vs. 51.8%, *p* = 0.01). There were less older elders among the empty nest ones (11.1% vs 19.2%, *p* = 0.04). The non-empty nest elders were more likely to utilize the family practice (54.8% vs 40.5%, *p* = 0.01).

**Table 1:** characteristics of elders reporting illness or discomfort in the previous two weeks

variables		Total		Non-NCD elders		NCD elders		Non-empty nest		Empty nest	
		*N* = 330		*N* = 85		*N* = 245		*N* = 177		*N* = 153	
		n	%	n	%	n	%	n	%	n	%
Socio-demographic status											
Gender	men	156	47.3%	35	41.2%	121	49.4%	79	44.6%	77	50.3%
	women	174	52.7%	50	58.8%	124	50.6%	98	55.4%	76	49.7%
Age	60–74	279	84.5%	68	80.0%	211	86.1%	143	80.8%	136	88.9%
	≥ 75	51	15.5%	17	20.0%	34	13.9%	34	19.2%	17	11.1%
Poverty	yes	103	31.2%	23	27.1%	80	32.7%	53	29.9%	50	32.7%
	no	227	68.8%	62	72.9%	165	67.3%	124	70.1%	103	67.3%
Empty-nest	yes	153	46.4%	43	50.6%	110	44.9%	–	–	–	–
	no	177	53.6%	42	49.4%	135	55.1%	–	–	–	–
Illiteracy	yes	143	43.3%	36	42.4%	107	43.7%	81	45.8%	62	40.5%
	no	187	56.7%	49	57.6%	138	56.3%	96	54.2%	91	59.5%
Health status											
Chronic diseases	yes	245	74.2%	–	–	–	–	135	76.3%	110	71.9%
	no	85	25.8%	–	–	–	–	42	23.7%	43	28.1%
Health behaviors											
Self-care in TCM	yes	211	63.9%	44	51.8%	167	68.2%	112	63.3%	99	64.7%
	no	119	36.1%	41	48.2%	78	31.8%	65	36.7%	54	35.3%
Drinking alcohol	yes	71	21.5%	16	18.8%	55	22.4%	36	20.3%	35	22.9%
	no	259	78.5%	69	81.2%	190	77.6%	141	79.7%	118	77.1%
Health service											
Distance^a^	< 1 km	190	57.6%	49	57.6%	141	57.6%	104	58.8%	86	56.2%
	2-3 km	103	31.2%	26	30.6%	77	31.4%	59	33.3%	44	28.8%
	> 3 km	37	11.2%	10	11.8%	27	11.0%	14	7.9%	23	15.0%
Family practice^b^	yes	159	48.2%	37	43.5%	122	49.8%	97	54.8%	62	40.5%
	no	171	51.8%	48	56.5%	123	50.2%	80	45.2%	91	59.5%

^a^Distance to the nearest medical institution

^b^Utilization of family practice service

### Prevalence of self-treatment and bivariate analysis

The prevalence of self-treatment was 35.2% (95% CI 30.0, 40.6%) among the subjects (Table 2). In addition, the prevalence of untreated cases was 5.0%. The most common self-treated illnesses included cold, hypertension, intervertebral disk disease and unknown illness (symptoms or discomfort of which the diagnosis or reasons remain unknown), of which the rates of self-treatment were 41.6, 24.0, 27.9 and 55.0%, respectively.

**Table 2:** prevalence and prevalence ratios of self-treatment regarding associated factors

Variables		Prevalence of self-treatment % (95% CI)	Bivariate analysis		Multiple regression	
			OR	95% CI	OR	95% CI
Elders in Sinan County		35.2 (30.0, 40.6)				
Socio-economic status						
Gender	men	28.8 (21.9, 36.6)	1.00		1.00	
	women	40.8 (33.4, 48.5)	1.70	(1.07,2.69)*	1.68	(0.95,2.97)
Age	60–74	33.3 (27.8, 39.2)	1.00		1.00	
	≥ 75	45.1 (31.1, 59.7)	1.64	(0.90,3.01)	1.71	(0.87,3.35)
Poverty	no	38.8 (32.4, 45.4)	1.00		1.00	
	yes	27.2(18.9, 36.8)	0.59	(0.35,0.98)*	0.69	(0.40,1.18)
Empty-nest	no	36.2 (29.1, 43.7)	1.00		1.00	
	yes	34.0 (26.5, 42.1)	0.91	(0.58,1.43)	0.94	(0.57,1.55)
Illiteracy	no	33.2 (26.5, 40.4)	1.00		1.00	
	yes	37.8 (29.8, 46.2)	1.22	(0.77,1.93)	0.88	(0.51,1.53)
Health status						
Chronic diseases	no	43.5 (32.8, 54.7)	1.00		1.00	
	yes	32.2 (26.4, 38.5)	0.61	(0.37,1.02)	0.87	(0.50,1.52)
TTO-value			4.73	(1.54,14.45)**	6.75	(1.93,23.60)**
Health behaviors						
Self-care in TCM	no	42.0 (33.0, 51.4)	1.00		1.00	
	yes	31.3 (25.1, 38.0)	0.63	(0.39,1.00)*	0.67	(0.40,1.12)
Drinking alcohol	no	39.4 (33.4, 45.6)	1.00		1.00	
	yes	19.7 (11.2, 30.9)	0.37	(0.20,0.71)**	0.42	(0.21,0.83)*
Health service						
Distance^a^	< 1 km	36.3 (29.5, 43.6)	1.00		1.00	
	2-3 km	34.0 (24.9, 44.0)	0.90	(0.55,1.49)	0.78	(0.45,1.35)
	> 3 km	32.4 (18.0, 49.8)	0.84	(0.40,1.78)	0.84	(0.39,1.86)
Family practice^b^	no	40.4 (32.9, 48.1)	1.00		1.00	
	yes	29.6 (22.6, 37.3)	0.62	(0.39,0.98)*	0.59	(0.36,0.96)*

^a^Distance to the nearest medical institution

^b^Utilization of family practice service

*P < 0.05; **P < 0.01

Across the whole sample, the prevalence of self-treatment regarding variables and results of the bivariate analysis are presented in Table 2. The female elders were more likely to self-treat their illness (OR 1.70; 95% CI 1.07–2.69). The population groups of lower proportion of self-treatment included elders in poverty (OR 0.59, 95% CI 0.35–0.98), elders with recent alcohol consumption (OR 0.37, 95% CI 0.20–0.71) and elders who utilise family practice services (OR 0.62, 95% CI 0.39–0.98). Elders who have higher TTO-value are more likely to self-treat (OR = 4.73, 95% CI 1.54–14.45).

However, the variables with significant differences in rates of self-treatment varied among different subgroups. No significant determinants were observed in the subgroup of the elders without chronic diseases, while the results in the group of elders with chronic disease were similar to those in the whole sample groups (Table 3). In the subgroup of non-empty-nest elders, the older elders (no less than 75 years old) were more likely to self-treat (OR 3.10, 95% CI 1.33–7.22) (Table 4). In the subgroup of empty-nest elders, the proportion of elders who self-treated was lower in those with chronic diseases than their counterpart (OR 0.47, 95% CI 0.23–0.98) (Table 4).

**Table 3:** Comparison of prevalence and prevalence ratios between elders with and without chronic diseases

variables		Non-NCD group					NCD population				
		Prevalence of self-treatment % (95%CI)	Bivariate analysis		Multiple regression		Prevalence of self-treatment % (95%CI)	Bivariate analysis		Multiple regression	
			OR	95% CI	OR	95% CI		OR	95% CI	OR	95% CI
Socio-economic status											
Gender	men	34.3 (19.1, 52.2)	1.00		1.00		27.3 (19.6, 36.2)	1.00		1.00	
	women	50.0 (35.5, 64.5)	1.92	(0.79,4.67)	1.72	(0.50,5.91)	37.1 (28.6, 46.2)	1.57	(0.92,2.70)	1.76	(0.90,3.43)
Age	60–74	41.2(29.4, 53.8)	1.00		1.00		30.8 (24.6, 37.5)	1.00		1.00	
	≥ 75	52.9 (27.8, 77.0)	1.61	(0.55,4.68)	1.31	(0.38,4.52)	41.2 (24.6, 59.3)	1.57	(0.75,3.30)	2.03	(0.88,4.68)
Poverty	no	45.2 (32.5, 58.3)	1.00		1.00		36.4 (29.0, 44.2)	1.00		1.00	
	yes	39.1 (19.7, 61.5)	0.78	(0.29,2.07)	0.76	(0.24,2.40)	23.8 (14.9, 34.6)	0.55	(0.30,1.00)*	0.64	(0.33,1.22)
Empty-nest	no	40.5 (25.6, 56.7)	1.00		1.00		34.8 (26.8, 43.5)	1.00		1.00	
	yes	46.5 (31.2, 62.3)	1.28	(0.54,3.02)	1.52	(0.56,4.12)	29.1 (20.8, 38.5)	0.76	(0.45,1.32)	0.70	(0.38,1.29)
Illiteracy	no	42.9 (28.8, 57.8)	1.00		1.00		29.7 (22.2, 38.1)	1.00		1.00	
	yes	44.4(27.9, 61.9)	1.07	(0.45,2.54)	0.66	(0.20,2.16)	35.5 (26.5, 45.4)	1.30	(0.76,2.23)	0.95	(0.50,1.83)
Health status											
Chronic diseases	no	–	–		–		–	–		–	
	yes	–	–	–	–	–	–	–	–	–	–
TTO-value		–	1.16	(0.14,9.56)	1.07	(0.10,11.44)	–	6.78	(1.73,26.63)**	13.56	(2.81 to 65.39)**
Health behaviors											
Self-care in TCM	no	43.9 (28.5, 60.3)	1.00		1.00		41.0 (30.0, 52.7)	1.00		1.00	
	yes	43.2 (28.3, 59.0)	0.97	(0.41,2.29)	1.03	(0.38,2.82)	28.1 (21.5, 35.6)	0.56	(0.32,0.99)*	0.59	(0.32,1.09)
Drinking alcohol	no	47.8 (35.6, 60.2)	1.00		1.00		36.3 (29.5, 43.6)	1.00		1.00	
	yes	25.0 (7.3, 52.4)	0.36	(0.11,1.24)	0.36	(0.09,1.56)	18.2 (29.5, 43.6)	0.39	(0.18,0.82)*	0.41	(0.18,0.91)*
Health service											
Distance^a^	< 1 km	40.8 (27.0, 55.8)	1.00		1.00		34.8 (26.9, 43.2)	1.00		1.00	
	2-3 km	53.8 (33.4, 73.4)	1.69	(0.65,4.41)	1.51	(0.54,4.22)	27.3 (17.7, 38.6)	0.70	(0.38,1.30)	0.55	(0.28,1.10)
	> 3 km	30.0 (6.7, 65.2)	0.62	(0.14,2.70)	0.60	(0.11,3.19)	33.3 (16.5, 54.0)	0.94	(0.39,2.25)	0.83	(0.33,2.10)
Family practice^b^	no	45.8 (31.4, 60.8)	1.00		1.00		38.2 (29.6, 47.4)	1.00		1.00	
	yes	40.5 (24.8, 57.9)	0.81	(0.34,1.92)	0.85	(0.33,2.17)	26.2 (18.7, 35.0)	0.57	(0.33,0.99)*	0.49	(0.27,0.89)*

^a^Distance to the nearest medical institution

^b^Utilization of family practice service

*P < 0.05; **P < 0.01

**Table 4:** Comparison of prevalence and prevalence ratios between non-empty-nest and empty-nest elders

variables		Non-empty nest					Empty nest				
		Prevalence of self-treatment % (95%CI)	Bivariate analysis		Multiple regression		Prevalence of self-treatment % (95%CI)	Bivariate analysis		Multiple regression	
			OR	95% CI	OR	95% CI		OR	95% CI	OR	95% CI
Socio-economic											
Gender	men	29.1 (19.4, 40.4)	1.00		1.00		28.6 (18.9, 40.0)	1.00		1.00	
	women	41.8 (31.9, 52.2)	1.75	(0.93,3.29)	1.52	(0.67,3.39)	39.5 (28.4, 51.4)	1.63	(0.83,3.20)	1.98	(0.84,4.64)
Age	60–74	31.4 (24.0, 39.8)	1.00				35.3 (27.3, 43.9)	1.00		1.00	
	≥ 75	55.8 (37.9, 72.8)	2.76	(1.29,5.92)**	3.10	(1.33,7.22)**	23.5 (6.8, 49.9)	0.56	(0.17,1.83)	0.43	(0.11,1.68)
Poverty	no	39.5 (30.9, 48.7)	1.00				37.9 (28.5, 48.0)	1.00		1.00	
	yes	28.3 (16.8, 42.3)	0.60	(0.30,1.21)	0.76	(0.35,1.63)	26.0 (14.6, 40.3)	0.57	(0.27,1.22)	0.68	(0.29,1.59)
Empty-nest	no	–	–		–		–	–		–	
	yes	–	–	–	–	–	–	–	–	–	–
Illiteracy	no	30.2 (21.3, 40.4)	1.00		1.00		36.3 (26.4, 47.0)	1.00		1.00	
	yes	43.2 (32.2, 54.7)	1.76	(0.95,3.26)	1.15	(0.54,2.47)	30.6 (19.6, 43.7)	0.78	(0.39,1.55)	0.54	(0.22,1.33)
Health status											
Chronic diseases	no	40.5 (25.6, 56.7)	1.00		1.00		46.5 (31.2, 42.3)	1.00		1.00	
	yes	34.8 (26.8, 43.5)	0.79	(0.38,1.60)	1.37	(0.60,3.15)	29.1 (20.8, 38.5)	0.47	(0.23,0.98)*	0.55	(0.24,1.23)
TTO-value			4.46	(1.04,19.16)*	9.20	(1.73,48.75)**		5.05	(0.90,28.51)	4.50	(0.61,33.04)
Health behaviors											
Self-care in TCM	no	38.5 (26.7, 51.4)	1.00		1.00		46.3 (32.6, 60.4)	1.00		1.00	
	yes	34.8 (26.1, 44.4)	0.85	(0.45,1.61)	0.89	(0.43,1.86)	27.3 (18.8, 37.1)	0.44	(0.22,0.87)*	0.39	(0.18,0.86)*
Drinking alcohol	no	39.7 (31.6, 48.3)	1.00		1.00		39.0 (30.1, 48.4)	1.00		1.00	
	yes	22.2 (10.1, 39.15)	0.43	(0.18,1.02)	0.48	(0.19,1.22)	17.1 (6.6, 33.6)	0.32	(0.12,0.84)*	0.28	(0.09,0.82)*
Health service											
Distance^a^	< 1 km	37.5 (28.2, 47.5)	1.00		1.00		34.9 (24.9, 45.9)	1.00		1.00	
	2-3 km	35.6 (23.6, 49.1)	0.92	(0.47,1.79)	0.87	(0.42,1.81)	31.8 (18.6, 47.6)	0.87	(0.40,1.89)	0.87	(0.35,2.16)
	> 3 km	28.6 (8.4, 58.1)	0.67	(0.20,2.27)	0.73	(0.20,2.63)	34.8 (16.4, 57.3)	1.00	(0.37,2.62)	1.00	(0.35,2.92)
Family practice^b^	no	41.3 (30.4, 52.8)	1.00		1.00		39.6 (29.5, 50.4)	1.00		1.00	
	yes	32.0 (22.9, 42.2)	0.67	(0.36,1.24)	0.65	(0.33,1.26)	25.8 (15.5, 38.5)	0.53	(0.26,1.08)	1.16	(0.08,16.51)

^a^Distance to the nearest medical institution

^b^Utilization of family practice service

*P < 0.05; **P < 0.01

### Logistic regression

After adjustments, in the whole sample group, higher TTO value (OR 6.75, 95% CI 1.93–23.60) were positively associated with self-treatment with larger effect size, while the habit of drinking alcohol (OR 0.42, 95% CI 0.21–0.83) and the utilization of family practice (OR 0.59, 95% CI 0.36–0.96) were negatively associated with self-treatment (Table 2).

However, when analysed in different subgroups, the significant predictors were somewhat different. The results of the elders with and without chronic diseases were presented in Table 3. After adjustment, in the subgroup with chronic diseases, the significant predictors included TTO values (OR 13.56, 95% CI 2.81–65.39), alcohol consumption (OR 0.41; 95% CI 0.18–0.91) and utilisation of family practice (OR 0.49, 95% CI 0.27–0.89). No significant variables were independently associated with self-treatment in the subgroup of the elders without chronic disease. As presented in Table 4,, having habits of self-care in TCM (OR, 0.39; 95% CI, 0.18–0.86) and recent alcohol consumption (OR, 0.28; 95% CI, 0.09–0.82) were found to be associated with a lower probability of self-treatment among the empty-nest elders. In the group of non-empty-nest elders, the older elders (OR 3.10; 95% CI 1.33–7.22), as well as those in better health status-higher TTO values (OR 9.20; 95% CI 1.73–48.75), were more likely to self-treat their illness (Table 4).

## Discussion

### Main results

The present study aimed to explore the determinants associated with self-treatment behaviour among the elderly, focusing on multi-dimensional factors in the rural China context. We found that 35.2% of the elders with illness in the previous 2 weeks practised self-treatment. The adjusted model showed that self-treatment was strongly associated with better health status, no recent alcohol consumption and no utilisation of family practice across the whole sample. However, the predictors, as well as their effect size, differed after stratifying the analysis into different subgroups.

### Discussion of the main results

#### Prevalence of self-treatment

This study showed that 35.2% of the subjects practised self-treatment. According to the 2013 report of CNHS, the prevalence of self-treatment was much higher (47.2%) among the elders in western rural China [13]. The proportion of untreated elders is 4.5%, which is similar to the result of CNHS (1.7% in western rural China, 2013) [13]. The decline of the prevalence of self-treatment may be partly due to the poverty reduction policies in poverty-stricken areas in the past few years [31]. The poverty reduction policies were found to be related to the increased outpatient rates and the inpatient rate [35, 36]. Another study conducted in Shanxi, a province in northwestern China in 2016, indicated that 79.4% of the middle-aged and elderly people had practised self-treatment 12 months before the survey [8]. Most previous studies focused on the behaviour of self-medication and found that the rates of self-medication among the elderly range from 20 to 60% [9]. The difference in methodology, especially the definition of self-treatment and self-medication, might account for the deviation in values to some extent. The differences in the contents of self-treatment/self-medication, as well as the diversity of the period of time considered, would result in the differences in the estimation of the prevalence [9, 37].

#### Predictors of the whole sample group

The prevalence rate of self-treatment was lower among the elders in poverty in the present study. It has been found that elders in middle income group are more likely to self-treat with over-the-counter (OTC) drugs than those in the lowest and highest income groups, which suggested that the elders in China with higher purchasing power preferred for timely self-initiated treatment strategies [38]. One of the possible explanations for the lower possibility of self-treatment for the poor population may be their poor affordability for self-purchased drug with out-of-pocket money, which cannot be covered by the medical insurance [39]. However, the limitation of the generalizability should be acknowledged, because this definition of poverty cannot be applied in the settings in other countries.

The utilisation of family practice services is strongly associated with the lower possibility of self-treatment in the adjusted model. A cross-sectional study in Spain indicated that visits to the family doctor or other specialists in the last 4 weeks are negatively related to self-treatment, especially in the age group of 45–74 years [37]. It was indicated in a study in China that residents who availed of family practice services perceive higher quality of primary care, including the aspect of first contact-access, continuity, comprehensiveness and coordination [18]. The utilisation of family practice may facilitate rural elders to the necessary medical services and reduce their resorting to self-treatment. However, according to the description analysis of the sample characteristics, only half of elders reported the utilisation of the services in the family practice before. The promotion of family practice is worthy of attention in remote areas.

Better health status is also related to the self-treatment behaviour in the present study, not only for the whole sample, but also in some of the subgroups. Some studies indicated that the prevalence of self-medication is higher among adults with poor self-rated physical and mental health [40], which is different from the result in our study. One of the possible explanations in the rural China context is that the people in remote rural areas are less likely to seek professional medical services if the illness or injury is not severe because of the difficulties of accessing and affording medical service [41]. Thus, rural elders might resort to self-treatment when in a relatively stable health condition, especially in the subgroups of the elders with long-term illness (chronic diseases) and the elders living with the family caregivers (non-empty nest). However, the large 95% CI of this variable implied that further studies with larger sample sizes were needed to generate the conclusion with higher precision.

The negative association between recent alcohol consumption and self-treatment also contradicted the results of previous studies at home and abroad. The data of the CNHS in 2008 reported a slightly higher proportion of self-treatment among those with drinking habits [17]. The middle-aged and elderly (45–74 years) with recent alcohol consumption in Spain are likely to resort to self-medication [37]. An empirical study in Finland showed that 19.2% of elders aged over 75 years old use alcohol as self-medication to cope with the heart and vascular disorders, sleep disorders, mental problems and other illnesses [42]. Some studies in alcohol consumption behaviour attributed the difference in drinking patterns to cultural aspects [43]. On the other hand, the cultural factors also played a role in the health-seeking behaviours. Many religious groups reject some or all mainstream health care, such as the Amish, Christian Science, Jehovah’s Witness and Islam [44]. Therefore, it can be inferred that there may be some confounding factors related to the subjects’ cultural background since our study was conducted in a remote multi-ethnic county.. There was a branch of Islamic faith-Hui people, living in the investigated county, and they have the cultural practice of abstinence from alcohol [45]. nA study in America illustrated that Muslims in the United States interpret their illness as part of God’s will, which serves as a barrier for them in seeking medical care [46]. Thus, the cultural background may partly explain the high proportion of self-treatment among elders without the habit of drinking alcohol, who may be the Muslim with specific health beliefs. Further studies were needed to illustrate the relationship between religious beliefs and self-treatment behaviour among the elders.

#### Comparison between subgroups of elders with and without chronic diseases

The prevalence of self-reported chronic diseases among the subjects was 74.2%, which was slightly higher than the rate of chronic disease reported in the CNHS in 2013 (65.6% of the elders aged over 65 in rural areas) [13]. It should be noted that, according to the definition, some of the elders with chronic diseases but in a relatively stable status that needed no any treatment or rest were not included in the population group of the presence of the illness or discomfort in the previous 2 weeks.

The prevalence rate of self-treatment was lower among the elders with chronic diseases, which differed from most previous studies. It was argued in the previous study that people with chronic diseases need regular follow-up visits and usually consume drugs with prescriptions [47]. The difference in prevalence was also not significant in the adjusted model. More empirical studies are needed to illustrate the association.

No significant predictors were found in the model of the elders without chronic diseases. A possible explanation is that the self-treatment for acute diseases is common across the population groups with low self-perceived health risks, including the people without chronic diseases [48, 49]. In addition, it should be noted that the small sample size (*N* = 85) in this subgroup may lead to the inadequate power of test, which might result in the false negatives of some variables [50]. Although there was no significant association, the effect size of the variable of gender was similar to that in the whole sample group, and empty-nest elders had higher possibility of self-treatment. Further researches are needed to examine the potential determinants.

As for the subgroup of elders with chronic disease, the risk factors are similar to those of the whole sample, which were discussed above. However, the association was stronger between the utilisation of family practice and the less likelihood of self-treatment, which implied that the family practice utilisation may benefit the elders with chronic disease more on the reduction of self-treatment behaviours. It was reported in a study conducted in southeastern China that among the residents having contracted with family physicians, those with chronic diseases were more likely to utilise the primary care as their usual source of care [18].

#### Subgroups of empty-nest and non-empty-nest elders

The most innovative design of this study is the illustration and the comparison between the risk factors of self-treatment among the empty-nest elders and the non-empty-nest elders. All independent predictors of self-treatment of empty-nest elders were in the category of health habits, including having the habits of self-care in TCM and no recent alcohol consumption. As for the self-care habit in TCM, on the one hand, people with the habits of self-care may have high perceived susceptibility and are likely to seek professional help instead of the risky self-treatment approaches. Some studies indicated that the self-care practice of some patients with chronic diseases is associated with the high perceived susceptibility of disorders [48, 49]. On the other hand, elders with the habits of self-care in TCM have higher affinities to TCM [34], and these elders tend to seek medical help in TCM sectors, in which they may be confronted with less barrier during the process of seeing and communicating with doctors compared to the western medicine sectors [51, 52], especially for empty-nest elders without support from younger family caregivers [12]. These findings may explain the lower rate of self-treatment among elders with higher affinities for TCM in this empty-nest subgroup.

As for non-empty-nest elders, those who were no less than 75-year-old or in better health status reported higher rates of self-treatment. The older elders are in the greater vulnerability of health and great need of care [53], and their family caregivers are often equipped with the knowledge of some common health problems and even some skills of healthcare tasks [54]. Given the long-term experience [37] of health problems and the inconvenience to visit doctors in remote areas [55], the older elders are likely to self-treat to cope with their discomforts with the support of their family caregiver, especially when they are in relatively stable health status.

### Implications for research and practice

The unequal prevalence of self-treatment in different population groups suggests the service gaps existing between the elders’ actual medical need and the health care delivery system among some older groups in local areas. The identification of the high-risk population of self-treatment would deepen our understanding of the needs of some key populations as well as the inequity in health care delivery among the elderly. Besides, the strong association between the utilisation of family practice services and the lower prevalence of self-treatment implies the potential of family practice in improving the accessibility of the medical help for the rural elders. Currently, the family practice system in China is still in its early stage [56]. A viable model of care [57] and more evidence-based practices are needed to improve the family practice system in China. The findings of this study may provide some clues for the redesign of the service. For example, the result of the subgroup of non-empty-nest elders suggested the substantial need of health education for family caregivers of the older elders, such as rational drug use, the training of necessary family caregiver skills and so on [58, 59].

However, appropriate self-treatment can bring convenience for individuals and help save medical cost [60], which will also benefit the healthcare system [61]. In consideration of the advantages and risks of self-treatment, as well as rising prevalence of long-term conditions, multiple medicines and increased health care utilisation, the roles of community pharmacists [62] and clinical pharmacists working within the family practice have received increasing interest [63]. Compared with some western countries, the pharmacist system in China is still underdeveloped [64], including the clinical pharmacists in family practice, which should also be attached importance to in developing improvement strategies for primary care.

In all, self-treatment is a critical issue for both health need and health service delivery. Further studies were needed to explore the self-treatment issue in different settings as well as the differences, such as the urban and rural areas, as well as the developed and under-developed areas.

### Limitations

Nevertheless, the study has several limitations that deserve to highlight. Firstly, the lack of standard definition for self-treatment makes the present results difficult to compare with previous findings [8, 9]. Secondly, the questionnaires did not contain more details of the subjects’ self-treatment behaviour, such as the specific form of self-treatment of each respondent, and the type of the medicine used in the self-treatment. Self-reported information may also lead to some reporting bias or recall bias because the majority of the respondents are elders [65]. In addition, the potential seasonal variation in healthy condition as well as in the health-seeking pattern is unavoidable, especially for the elders, which may limit the generalisability of the study.

## Conclusion

In general, self-treatment is a common behaviour among the elders in rural China. Stable health status, no recent alcohol consumption and no utilisation of family practice are associated with a higher probability of self-treatment among rural elders. Older elders in the non-empty nest group were more likely to self-treat, while the empty-nest elders with self-care habits in traditional Chinese medicine were less likely to self-treat. The findings suggest the potential of family practice in improving the access to medical care for rural elders. The identification of the determinants of some specific population groups may provide clues for the improvement strategies of healthcare delivery system for the rural elders. Further studies on self-treatment patterns and decision-making mechanisms are needed to elucidate the potential need and access barriers of healthcare among the rural elders.

## Data Availability

The datasets used and analysed during the current study are available from the corresponding author on reasonable request.

## Abbreviations

ADRs: Adverse drug reactions
CNHS: China National Health Survey
LMICs: Low and middle income countries
TCM: Traditional Chinese medicine
TTO: Time trade-off; OTC: over-the-counter
